# Hypoglycemia in a Non-diabetic Patient and the Side Effects of Diazoxide Use

**DOI:** 10.7759/cureus.36804

**Published:** 2023-03-28

**Authors:** Patrícia C Brito, Valentim Lopes, Eulália Antunes, Marina Alves, Inês Gonçalves, Ana Catarina Matos

**Affiliations:** 1 Department of Endocrinology, Hospital de Braga, Braga, PRT; 2 Department of Internal Medicine, Hospital de Braga, Braga, PRT

**Keywords:** hypoglycemia, insulinoma, diazoxide, medication side-effects, congestive heart failure

## Abstract

A low blood glucose level (less than 55 mg/dL) associated with autonomic and neuroglycopenic signs and symptoms that resolve after glucose administration establishes Whipple’s triad, indicating the presence of a hypoglycemic disorder. Insulinoma remains the most common cause of endogenous hyperinsulinemia.

We present the case of a 73-year-old male who was brought to the emergency department after losing consciousness. On initial assessment, severe hypoglycemia was identified and treated. No abnormalities were detected on the physical examination, initial blood tests, abdominal ultrasound and computed tomography (CT) thorax, and abdomen and pelvis. The patient had another episode of symptomatic hypoglycemia, and the blood tests performed were compatible with endogenous hyperinsulinism. The patient was started on diazoxide to prevent further hypoglycemia episodes. Magnetic resonance imaging (MRI) showed a nodular area in the cephalic region of the pancreas, and the patient was discharged with diazoxide and flash glucose monitoring. In the follow-up appointment, he presented with signs and symptoms of congestive heart failure. Endoscopic ultrasound was requested, but the patient was at high risk for complications while undergoing the procedure under anesthesia due to congestive heart failure. A ^68^Gallium-DOTA-NOC positron emission tomography and computed tomography (PET-CT) was requested and confirmed the presence of a nodular area in the cephalic region of the pancreas. He was referred to general surgery for definitive treatment.

Insulinoma is still a challenging medical condition. Therefore, management by a multidisciplinary team is essential. This case highlights the impact that side effects of medication used to treat this condition can have. Diazoxide was initiated to stop severe recurrent hypoglycemia; however, the patient developed congestive heart failure and was unable to undergo an endoscopic ultrasound to localize the lesion, resulting in a delay in diagnosis and definitive treatment. Diazoxide is a potent hyperglycemic drug but it can also cause fluid retention, nausea, hypertrichosis, neutropenia, and thrombocytopenia.

## Introduction

A low blood glucose level (less than 55 mg/dL) associated with autonomic (tremors, sweating, and palpitations) and neuroglycopenic (confusion, dysarthria, visual impairment, or even coma) signs and/or symptoms that resolve after glucose administration establishes the Whipple’s triad, indicating the presence of a hypoglycemic disorder [1-4]. Hypoglycemia in non-diabetic patients is rare [5] and the diagnostic cascade can be challenging.

There are numerous causes of hypoglycemia. Medication is a common one and insulin or insulin secretagogues (for example, sulfonylureas) should always be considered even in non-diabetic patients, as well as other glucose-lowering drugs (beta-blockers, lithium and fluoroquinolones, among others). Furthermore, it is important to exclude acute alcoholism, critical illness, liver/kidney failure, cortisol deficiency, gastric/bariatric surgery, insulinoma, and islet cell hyperplasia (including nesidioblastosis). Other causes, even more rare, include genetic (monogenic congenital hyperinsulinism, insulin receptor mutations, and inborn errors of metabolism), paraneoplastic (non-islet-cell tumors), and autoimmune (Hirata’s disease and Flier’s syndrome) [1-6]. Therefore, an exhaustive clinical history, including the time of onset of symptoms and its correlation with meals, and examination are essential.

Insulinoma is the most common cause of endogenous hyperinsulinism, although it has an annual incidence of only four in one to three million people [1,3,5,7,8]. It is generally associated with fasting hypoglycemia, and the diagnosis is established when there is biochemical evidence of hypoglycemia with an inappropriately normal/high level of insulin (equal to or greater than 3 μU/mL) and C-peptide (equal to or greater than 0.6 ng/mL) occurring spontaneously or during a 72-h fasting test. Beta-hydroxybutyrate is low (equal to or less than 2.7 mmol/L) even with the prolonged fast because insulin has an anti-ketogenic effect [3,6].

About 90% of insulinomas are benign, and they are usually single tumors of the pancreas. Therefore, surgery can be curative if the tumor can be completely removed. There are also medical treatment options for those who are not good candidates for surgical excision [3,7-9].

Localizing the lesion can be difficult because it is often small (with less than 2 cm), although this is essential to define the best surgical approach [3,10]. Ultrasound, computed tomography (CT) scan, and magnetic resonance imaging (MRI) are non-invasive and widely available methods. If the tumor is not found with these modalities, endoscopic ultrasound detects 70-95% of insulinomas and intra-arterial calcium stimulation with hepatic venous sampling more than 80%. However, these techniques are invasive, have high costs and require an experienced interventional radiologist. Another option to localize these lesions is ^68^Gallium-labeled somatostatin analog positron emission tomography (PET) with combined CT [8].

## Case presentation

We present the case of a 73-year-old male that was brought to the emergency department after losing consciousness. On initial assessment, severe hypoglycemia (24 mg/dL) was identified, and the patient was treated with 40 mL of 30% dextrose by the emergency medical team. The patient regained consciousness afterward.

Upon taking a history, the patient and his wife reported two episodes of dizziness in the last month with associated sweating and dysarthria that they thought to be related to low blood pressure. These episodes resolved when he drunk a glass of water with sugar and occurred in the morning, although the patient did not recall if it was before or after breakfast. He had a history of hypertension and dyslipidemia. He denied any history of diabetes or taking any glucose-lowering medication. His medication regimen included amlodipine, olmesartan, hydrochlorothiazide, and simvastatin. He had never smoked and drank 12 g of alcohol per day. There was no relevant family history, including no history of autoimmune diseases.

Upon arrival at the emergency department, blood pressure was 132/69 mmHg, heart rate was 74 beats per minute, oxygen saturation was 98% on room air, temperature was 36.9°C, and glycemia was 100 mg/dL. He was alert, oriented to person, space and time, cooperative and had no motor/sensitive deficits. No abnormalities were detected on the remaining physical examination.

Analytically, he had a complete blood count with no abnormalities, creatinine level was 0.7 mg/dL, ionogram was within normal ranges, as well as aspartate aminotransferase, alanine aminotransferase, gamma-glutamyl-transferase, alkaline phosphatase, and bilirubin. The thyroid function was normal. An abdominal ultrasound and a CT thorax, abdomen and pelvis were performed and did not reveal any abnormalities, including pancreatic masses. He was admitted to the hospital for observation and further investigation.

On the second day of observation, the patient had another episode of symptomatic hypoglycemia despite fluid therapy and a personalized diet, during which blood tests were performed. Laboratory results revealed a blood glucose of 52 mg/dL, with an insulin level of 8.02 μU/mL and a C-peptide level of 1.14 ng/mL. According to the Endocrine Society guidelines “Evaluation and Management of Adult Hypoglycemic Disorders: An Endocrine Society Clinical Practice Guideline,” these results are compatible with endogenous hyperinsulinism. Glycated hemoglobin (HbA1c) was 4.8%, and cortisol level at 8 am was 17.07 μg/dL (N: 4.3-22.4 μg/dL) (Table 1). Thus, an abdominal MRI was required to evaluate the presence of pancreatic masses.

**Table 1:** Laboratory findings during a hypoglycemic episode. ^a^According to the Endocrine Society guidelines “Evaluation and Management of Adult Hypoglycemic Disorders: An Endocrine Society Clinical Practice Guideline.”

	Glucose (mg/dL)	Insulin (μU/mL)	C-peptide (ng/mL)	HbA1c	Cortisol (8 am) (μg/dL)
Normal range	>55	3-25	0.81-3.85	-	4.3-22.4
Value found in the essay	52	8.02	1.14	4.8%	17.07
Reference range for the diagnosis of insulinoma^a^	<55	≥3	≥0.6	-	-

**Figure 1:**
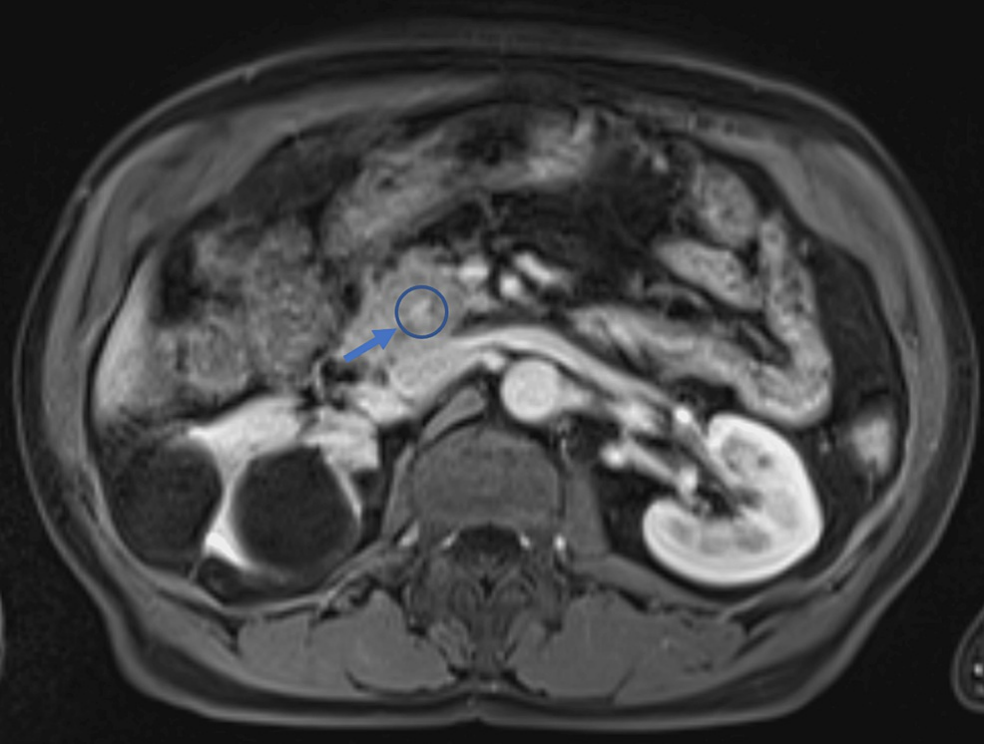
MRI showing a nodular area in the cephalic region of the pancreas with 10 mm that could represent a neuroendocrine tumor. MRI: magnetic resonance imaging.

Patient was started on diazoxide 100 mg twice a day to prevent further hypoglycemia while waiting for the results of the insulin antibodies and MRI. He had no further hypoglycemic episodes after diazoxide was introduced, and he had no immediate side effects. The MRI showed a nodular area in the cephalic region of the pancreas with 10 mm, which could represent a neuroendocrine tumor without any evidence of metastases, including hepatic metastases (Figure 1).

The patient was discharged with diazoxide 200 mg per day and with flash glucose monitoring. In the follow-up appointment a month later, he had had no further episodes of hypoglycemia, but he now presented with bilateral edema of the lower limbs associated with complaints of dyspnea on minor exertion, orthopnea, and the impression of decreased urinary output. Diagnosis of heart failure was made considering the presence of suggestive symptoms, an elevated value of N-terminal brain natriuretic peptide (NT-proBNP) (4300 pg/mL), and a previous transthoracic echocardiogram with evidence of concentric hypertrophy of the left ventricle with a fractional ejection fraction of 53%. Endoscopic ultrasound was requested (to confirm the presence of a pancreatic mass) before he was discharged, but the patient was considered to be at high risk for complications while undergoing the procedure under anesthesia due to signs and symptoms of congestive heart failure. He was referred to a cardiologist and started on 2.5 mg of bisoprolol and 80 mg of furosemide. We have managed to reduce the dose of diazoxide to 175 mg per day without new episodes of hypoglycemia. Since he could not undergo an endoscopic ultrasound, a ^68^Gallium-DOTA-NOC PET-CT was requested. The report confirmed the presence of a nodular area consistent with a neuroendocrine tumor with increased expression of somatostatin receptors. There was no evidence of metastases. After reducing the dose of diazoxide and starting a beta-blocker and a loop diuretic, heart failure signs/symptoms improved. He was referred to general surgery for definitive treatment.

## Discussion

This case highlights the impact that adverse side effects of medication can have on the patient’s ability to tolerate certain imaging methods to localize the tumor, which then causes a delay in definitive treatment. Diazoxide was initiated to stop severe recurrent hypoglycemia, but the patient developed congestive heart failure due to fluid retention. Due to this, he was unable to undergo an endoscopic ultrasound resulting in a delay in the diagnosis and therefore the definitive treatment.

Diazoxide is a potent hyperglycemic drug. It inhibits insulin secretion by opening the adenosine triphosphate (ATP)-dependent potassium channel of the beta cells, and it probably has an extra-pancreatic effect (enhancing glycogenolysis by inhibiting cyclic adenosine monophosphate phosphodiesterase) [3,11,12].

It also causes fluid retention, resulting in edema, mainly when administered in higher doses. The incidence of fluid retention secondary to diazoxide is variable, ranging between 31% and 83% [13-15]. The mechanisms behind this adverse effect are still under investigation but seem to be related to increased antidiuretic hormone secretion, alteration of intrarenal blood flow, and stimulation of the renal sympathetic nerve activity [13]. Thus, when starting treatment with diazoxide, patients should be carefully monitored for weight, electrolytes, edema, and cardiopulmonary function. To counterbalance this fluid retention effect, a thiazide diuretic (like hydrochlorothiazide) can be associated, with the advantage that hydrochlorothiazide synergizes with diazoxide in the hyperglycemic effect. When this strategy, ally with a reduction of the diazoxide dose (when possible), is not enough to treat the fluid retention, other drugs, namely, those usually used to treat congestive heart failure like loop diuretics, should be started [13].

Other possible adverse effects of diazoxide, that our patient did not develop, include nausea, hypertrichosis, neutropenia, and thrombocytopenia [3,12]. Other medical treatment options to treat hyperinsulinemia-related hypoglycemia include somatostatin analogs, glucocorticoids, calcium channel blockers, acarbose, everolimus, and lutetium [3,11,16]. Surgery remains the preferred option when there is a localized lesion, as it can be curative.

## Conclusions

Insulinoma is still a challenging medical condition, as its diagnosis requires the presence of Whipple's triad and measuring insulin and C-peptide levels during a hypoglycemic episode. Additionally, localizing insulinomas can be difficult, as most of these tumors are small and may not be detected by CT scans. Treating insulinoma can be challenging due to the potential adverse effects of treatment. Therefore, management by a multidisciplinary team is essential.
